# Diseases of the Blood

**Published:** 1905-07-08

**Authors:** 


### DISEASES OF THE BLOOD.

**Basophilic Granules in Erythrocytes.**—Cadwalader[1] has made a study of the basophilic granules in red-blood cells in lead-poisoning and other forms of anæmia. In healthy individuals it was found that red corpuscles containing basic-staining granules were present in from ·4 to 1·6 per cent. of the red corpuscles. In eleven lead-workers without subjective symptoms a slight reduction of both the hæmoglobin and the total number of red corpuscles was found; the number of granular red corpuscles was, on the average, 4·2 per cent., and a few nucleated red corpuscles were present. In actual cases of lead poisoning the number of granular cells was decidedly increased—averaging 11·5 per cent., and accompanying this increase there was in each case one or more nucleated red corpuscles. In a series of cases of anæmia, other than that caused by lead-poisoning, granular red corpuscles were invariably found and were increased slightly (average, 6·6 per cent.), while if the blood contained nucleated corpuscles the increase was very marked. The granules contained in the red corpuscles could be divided into three general classes—fine and coarse thread-like strands, dot-like granulations, and dense coarse masses. The first variety consisted of strands, one or more, often overlapping one another and suggesting a network: this type was seen in the blood of every case, whether normal or pathological. The second variety—dots of the size of an eosinophile granulation—was found in all cases of lead poisoning and occasionally in the primary anæmias. The third variety consisted of a dark blue staining mass about the size of the nucleus of a normoblast, generally in the centre of the cell, and was granular. These were seen especially where nucleated red corpuscles were present. These three varieties of granulations probably represent different stages in the dissolution of the nucleus of a red cell. In support of this opinion there are the facts that the staining properties of the granulations are similar to those of the nuclei of nucleated red cells, and that there is a constant association of granule-containing red cells and nucleated red cells; and in any one case their relative numbers vary inversely, an increase in the granular cells being synchronous with a decrease of nucleated red corpuscles. The granular cells are thus the results of a fragmentation of the nucleus of nucleated red cells.

**Splenic anæmia.**—Steugel,[2] who had previously expressed the conviction that under the name of splenic anæmia many different sorts of diseases have been classed, has recently published reports of five cases and a review of the subject. He says that many of the cases properly belong to one or other of the following conditions: 1. Progressive pernicious anæmia, or intense secondary anæmia. 2. Pseudo-leukæmia. 3. Leukæmia, in which subsidence of leukocytosis has, for the time, taken place. 4. Cirrhosis of the liver, with enlargement of the spleen; either from obstruction of the portal circulation or from the action of the irritant causing the cirrhosis. Such cases are probably rare. 5. Hypertrophic cirrhosis of the liver (Hanot's disease). 6. Chronic splenitis in children, due to rickets, congenital syphilis, or in the disease called "anæmia infantum pseudo-leukæmia" or "splenic anæmia" of children. Leaving out of consideration the cases of splenic enlargement which can be classified under the above heads, there remain certain groups in which the splenic disease is the predominating feature. Of such cases the following varieties may be recognised provisionally:—(1) *Simple splenomegaly.*—Anæmia is not by any means an invariable symptom, though usually present, especially when hæmorrhages occur. Pathologically speaking, this variety belongs to the category of chronic splenitis secondary to malaria, rickets, and chronic gastro-intestinal disease. The lesion is essentially a fibrous hyperplasia. (2) *Simple splenomegaly terminating in cirrhosis of the liver.*—It is possible that the cases which ultimately develop cirrhosis of the liver and assume the fully developed character of Banti's disease are cases which from the start had a prominent grade of endothelial proliferation in the splenic sinuses. 3. *Splenomegaly with marked Constitutional Disturbances.*—In a certain number of cases of splenomegaly there is a disturbance of the general health, causing a stunted growth, clubbed fingers, osseous changes, and pigmentation of the skin. The cases have mostly been observed in children or young persons. 4. *Splenomegaly Primitive* (*Gaucher's Type*).—While it is not possible to distinguish this variety clinically, there is a distinctive pathological change—the presence of irregular oval spaces surrounded with fibrous tissue and more or less filled with large endothelial cells. There are all grades, from one in which the predominating feature is the fibrous change to one where the macroscopic and microscopic features are those of an active neoplastic formation.

[1] Cadwalader, Amer. Jour. Med. Sc., February. [2] Stengel, Amer. Jour. Med. Sc., February.

(*To be continued.*)

